# Patient preferences concerning the efficacy and side-effect profile of schizophrenia medications: a survey of patients living with schizophrenia

**DOI:** 10.1186/s12888-018-1856-y

**Published:** 2018-09-12

**Authors:** Eric Achtyes, Adam Simmons, Anna Skabeev, Nikki Levy, Ying Jiang, Patricia Marcy, Peter J. Weiden

**Affiliations:** 1 Cherry Health and Michigan State University College of Human Medicine, Grand Rapids, MI USA; 2 Alkermes, Inc., Waltham, MA USA; 3 Vanguard Research Group, Northwell Health System, Manhasset, NY USA

**Keywords:** Adherence, Antipsychotics, Weight, Side effects

## Abstract

**Background:**

Despite the availability of numerous antipsychotic medications, many patients with schizophrenia continue to experience side effects that contribute to the overall burden of the illness. The present survey of patients with schizophrenia and schizoaffective disorder aimed to assess patient attitudes toward antipsychotic treatment, and understand key factors about willingness to try a new medication.

**Methods:**

A cross-sectional survey was administered to 250 patients with a primary clinical diagnosis of a schizophrenia spectrum disorder across five outpatient clinics in the United States. The survey included self-reported gender, age, weight, and height, and questions about the importance of efficacy and side effects on the decision to take a prescribed antipsychotic medication.

**Results:**

Patients rated efficacy and side effects as important attributes of antipsychotic treatment, with 93.6% and 83.6% of patients listing these as “very” or the “most” important factors in taking prescribed medication. A total of 87.6% of respondents identified the ability to think more clearly as an important property of their medication. Patients identified weight gain, physical restlessness, and somnolence as important side effects of current treatments (“very” or “most” important by 61.6%, 60.8%, and 58.8%, respectively). When asked about willingness to change antipsychotic medication, anticipated weight gain had a negative influence on willingness to try the new treatment, with 22.0% declining to try a medication that would lead to weight gain of 2.7–4.5 kg (6–10 lb), 34.0% declining for anticipated weight gain of 5.0–9.1 kg (11–20 lb), and 52.4% declining for anticipated weight gain greater than 9 kg (20 lbs).

**Conclusion:**

Patients living with schizophrenia spectrum disorders are influenced by many factors when considering whether to take their medication, including efficacy and side effects. It is important for clinicians to assess specific patient concerns to develop a comprehensive treatment plan that maximizes adherence to the prescribed therapy.

**Electronic supplementary material:**

The online version of this article (10.1186/s12888-018-1856-y) contains supplementary material, which is available to authorized users.

## Background

Despite improvements in the side-effect profile of antipsychotic medications brought on by the development of atypical antipsychotics, many patients with schizophrenia continue to experience side effects that contribute to the overall burden of the illness [1]. Historically, the most significant side effects, associated with typical antipsychotics, were movement disorders such as antipsychotic-induced Parkinsonism, dystonia, akathisia, and tardive dyskinesia [2, 3]. Although the introduction of atypical antipsychotics lowered the burden of movement disorders, other side effects such as sedation, weight gain, and metabolic dysregulation have become prominent and problematic [1–4]. Weight gain as a side effect is particularly significant in schizophrenia because of the dual challenges of patients’ reluctance to take or remain on a medication that causes weight gain, and of elevated cardiovascular risk in this population [4, 5]. The combined influence of inadequate treatment efficacy and side effects contributes to high rates of treatment discontinuation and frequent switching between medications in patients with schizophrenia [6]. In the Clinical Antipsychotic Trials of Intervention Effectiveness (CATIE) study of 1493 patients with schizophrenia, 74% discontinued their initially assigned study medication before 18 months [1].

Current strategies for the design of clinical trials in schizophrenia are based on clinically determined outcomes for efficacy and safety. However, many factors impact adherence to medication, including patient expectations, perceived benefit of treatment, current phase of illness, and the side-effect profile of current and past medications; particularly weight gain, movement disorders, and sedation [7, 8]. Formal randomized controlled trials that factor in these patient-reported outcomes are rare despite their importance in psychiatry because they allow subjective insight into the impact of a treatment on symptoms, tolerability to medication, and quality of life [9]. Therefore, to improve adherence, it is critical that the development of new treatments is based on efficacy and side-effect profiles that are deemed acceptable to patients.

The objective of this study was to evaluate the self-reported reasons for continuing or discontinuing antipsychotic medication in outpatients with schizophrenia spectrum disorders receiving services across five treatment sites in the United States. A survey was designed to ascertain the preferences of patients with schizophrenia spectrum disorders, evaluating the relative importance of medication efficacy and perceived side-effect burden and their impact on the patient’s decision to take antipsychotic medication.

## Methods

### Survey design

The survey was developed with input from the patient advocacy community and from patients living with schizophrenia to ensure the design of a short, easy-to-administer scale designed to assess patient preferences. Insights and feedback from mental health advocates and patients were sought to support the design of a questionnaire aimed at understanding patient preferences regarding the importance of common side effects in treatment decision-making. Feedback on the design and language of the initial draft of the questionnaire led to three valuable insights that were incorporated into the final version: 1) patients found the initial pilot survey too long and confusing, leading to a revised survey that was shortened to focus only on specific side effects judged to be common and associated with adherence challenges [10]; 2) the survey format, which was initially designed to include several question types, was deemed too complex; a straightforward and parallel-constructed Likert-type scale design was subsequently implemented; 3) the symptom descriptions (originally based on the Positive and Negative Syndrome Scale [PANSS]) were considered too technical and not easily understood (e.g., ‘Medicines that make the hallucinations or paranoia go away’); on that basis, wording changes suggested by the reviewers were incorporated.

The sample size (250 patients across five clinics) was selected from four states to achieve a geographically-by-region diverse representation. Five community clinics from four states provided a representation of urban (2), suburban (2), and rural (1) communities. Individual clinics used a sample of convenience based on patient flow in order to identify individuals willing to participate in the survey. All clinics were able to complete 25 to 75 surveys, between October 15, 2016 and December 21, 2016.

### Study design

This cross-sectional survey was administered to 250 patients aged ≥18 years with a primary clinical diagnosis of schizophrenia or schizoaffective disorder, according to the *Diagnostic and Statistical Manual of Mental Disorders* (Fifth Edition) (DSM-5) [11]. No additional inclusion or exclusion criteria were applied. Patients were recruited from five outpatient clinics across the United States, including community mental health clinics that provide primary treatment to patients with severe mental illness. Recruitment methods varied across clinics, but generally patients were approached at check-in or before meeting with their healthcare provider, if they met the diagnostic criteria. The paper-based survey was completed in the clinic by the patient with assistance from staff, if needed, and each patient was given a $25 gift card upon completion of the survey.

The survey included self-reported gender, age, weight, and height as well as six questions regarding the importance of efficacy and side effects when taking a prescribed medication. Here, findings from the first five items of the six item survey, pertaining to patient medication preferences, are reported. The final survey question is not reported as it is unrelated to that objective. The full questionnaire is provided in the Additional file 1.

### Data analysis

Data analysis of non-descriptive measurements was performed using SAS software (SAS Institute, Cary, NC).

## Results

### Patients

Two hundred and fifty patients completed the survey; of these, 64.0% were male (*n* = 160) and 2.0% did not include data on gender (*n* = 4). The mean age of the survey respondents was 43 years (range, 18–72 years), mean weight was 91 kg (200 lb) (range, 49–182 kg [107–402 lb]), and mean body mass index (BMI) was 30 kg/m^2^ (range, 15–63 kg/m^2^) (Table 1).

**Table 1:** Patient characteristics

Characteristics	*N* = 250
Age, years (range)	43 (18–72)
Gender, n (%)	
Male	160 (64)
Female	86 (34)
Missing gender data	4 (2)
Weight, total	
kg (range)	91 (49–182)
lb (range)	200 (107–402)
Weight, male	
kg (range)	92 (49–182)
lb (range)	204 (109–402)
Weight, female	
kg (range)	87 (49–159)
lb (range)	192 (107–350)
BMI, kg/m^2^ (range)	
Total population	30 (15–63)
Males	29 (15–51)
Female	33 (18–63)

All values are means

Abbreviation: *BMI* body mass index

### Key survey findings

Patients identified both efficacy and side effects as important attributes of medications for the treatment of schizophrenia. Based on a 5-point scale, from “I don’t have this problem” to “most important,” most patients reported the ability to think more clearly as an important reason to take their medication (87.6% rated it as “most important” or “very important”). Most patients also reported the ability to stop hallucinations or paranoia as important (76.4% rated it as “most important” or “very important”) and the ability to have fewer side effects than they experienced with their current treatment as important (71.6% rated it as “most important” or “very important”) (Survey Question 1).

When considering adherence to medication, efficacy and side effects were identified as the most important drivers for patients to take their prescribed medicine (93.6% and 83.6% rated it as “most important” or “very important,” respectively) (Table 2). The presence of active symptoms of schizophrenia was an important factor for 80.4% of patients. Slightly more than half the patients (54.8%) reported that someone reminding them to take their medication was “most important” or “very important,” and ease of medication administration was “most important” or “very important” for 67.6% of patients.

**Table 2:** Degree of helpfulness of a new medication for the treatment of patients with schizophrenia

Quality	*N*	Most important, %	Very important, %	Somewhat important, %	Not important, %
How well medication treats my schizophrenia	250	66.0	27.6	4.4	2.0
If I’m actively having symptoms	250	49.6	30.8	10.4	9.2
Side effects of medication	250	45.6	38.0	12.8	3.6
How easy the medication is to take	250	38.4	29.2	21.2	11.2
Somebody reminds me or gives me the medication	250	29.6	25.2	20.4	24.8

Source: Survey Question 4. When you are deciding whether to take a medicine that has been prescribed to you, how important is each of the following factors?

**Fig. 1:**
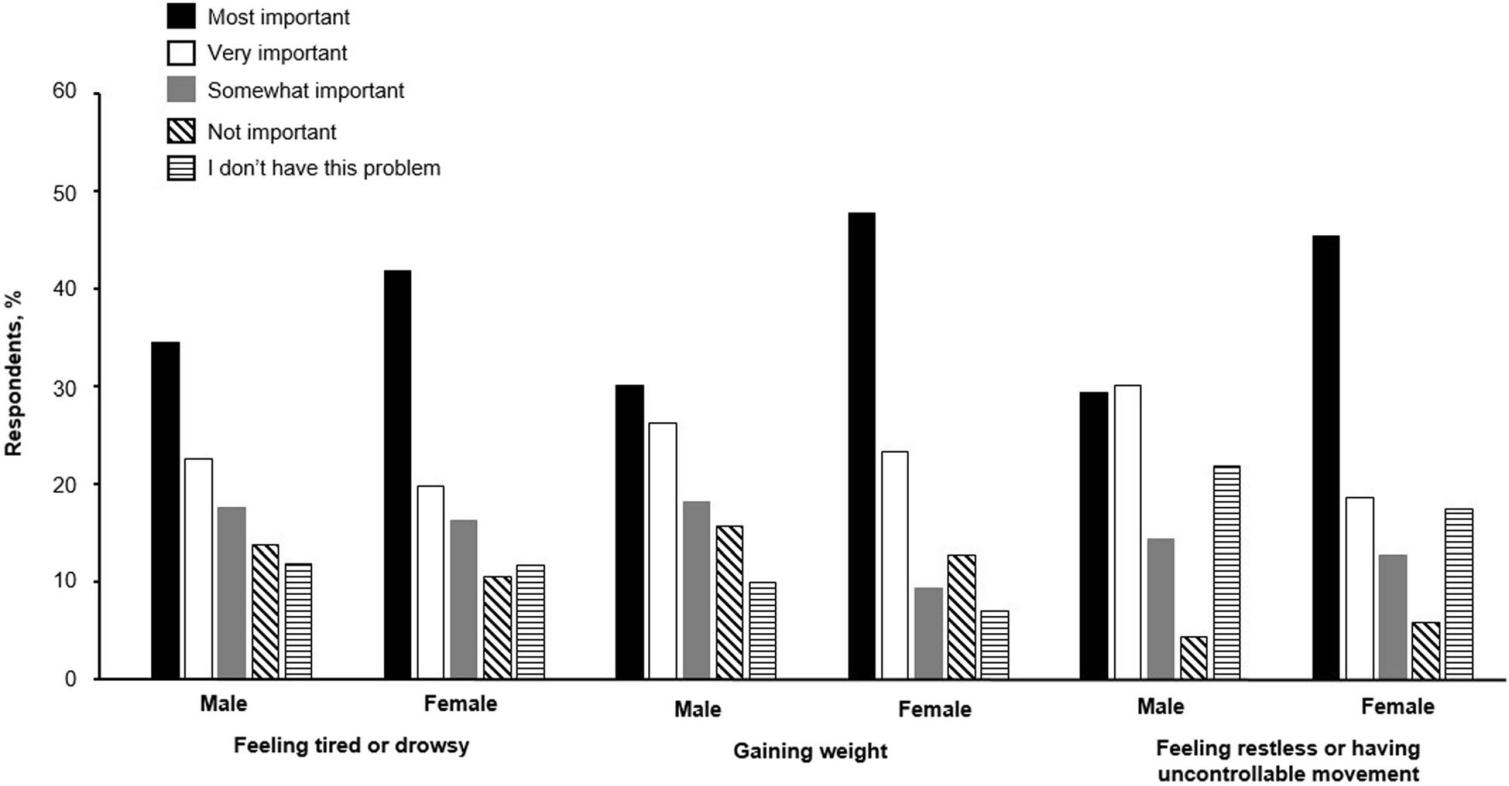
Importance of common side effects of schizophrenia treatments by gender*. Source: Survey Question 2. Many current treatments for schizophrenia have side effects. How important are these side effects to you? *Data not included for patients who did not self-report gender (*n* = 4)

When asked about the side effects of current treatments for schizophrenia, 61.6% of patients rated weight gain as “most important” or “very important”, compared with 60.8% for restlessness or akathisia and 58.8% for somnolence. The perceived importance of side effects of schizophrenia medication varied with gender; female patients rated all the side effects included in the survey as of greater importance than did male patients (Fig. 1). Considering the perceived importance of side effects according to gender, weight gain was considered to be “most important” or “very important” for 70.9% of female patients and 56.3% of male patients. Feeling tired or drowsy was viewed as being “most important” or “very important” for 61.6% of female patients and 56.9% of male patients. Female patients also assigned feeling restless or having uncontrollable movement slightly greater importance than did male patients (64.0% of females and 59.4% of males rated this as “most important” or “very important,” respectively) (Fig. 1).

**Fig. 2:**
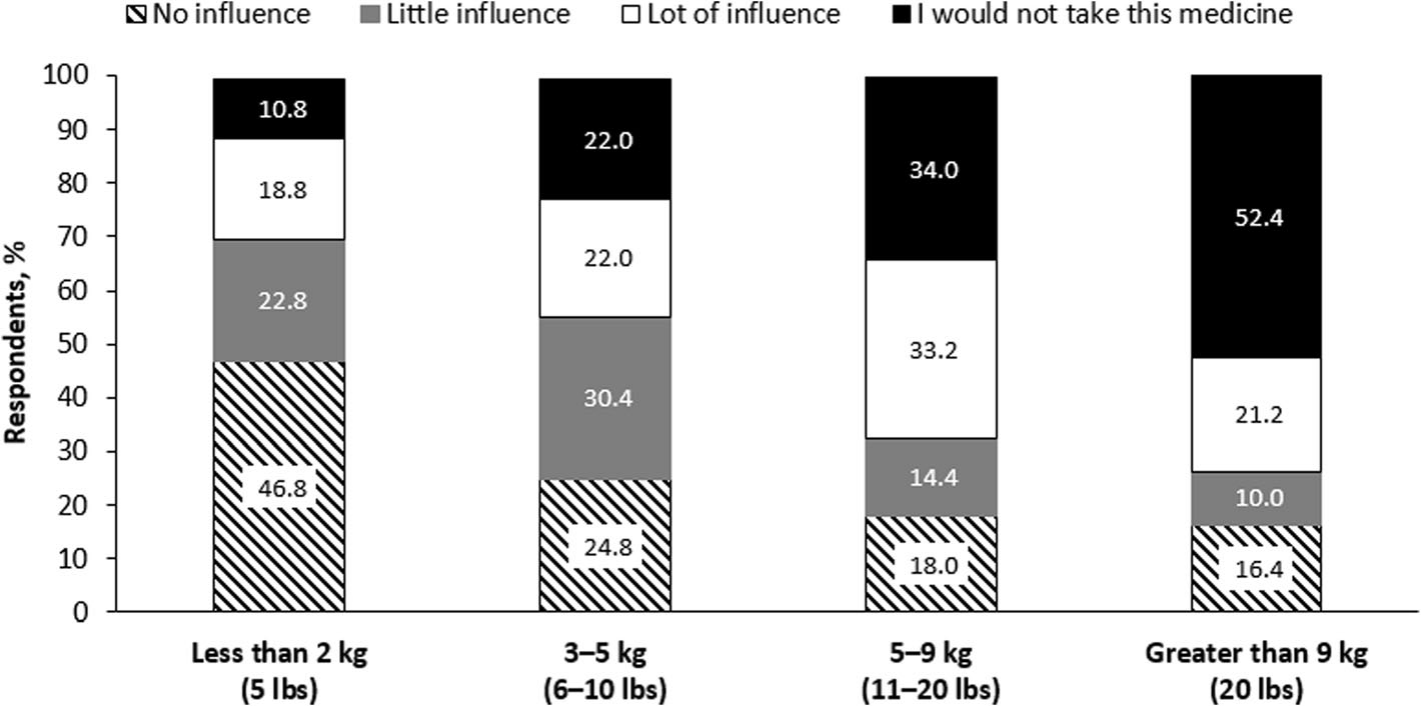
Influence of anticipated weight gain on patient’s decision to take medicine*. *The number (percentage) of patients with missing responses in each weight gain category are: less than 2 kg, *n* = 2 (0.8%); 3–5 kg, *n* = 2 (0.8%); 5–9 kg, *n* = 1 (0.4%). Source: Survey Question 3. One side effect of many medicines for schizophrenia is weight gain. How much would gaining weight influence your decision to take a medicine?

The presence of anticipated weight gain had a significant impact on self-reported willingness to accept a change in antipsychotic medication. The absolute amount of anticipated weight gain was associated with reluctance to change, with 73.6% of patients indicating that a weight gain of > 9 kg (> 20 lbs) “Would influence my decision a lot” or “I would not take this medicine” compared with 67.2% who were influenced by a weight gain of 5.0–9.1 kg (11–20 lb), 44.0% who were influenced by a weight gain of 2.7–4.5 kg (6–10 lb) and 29.6% who were influenced by a weight gain of < 2.3 kg (< 5 lb) (Fig. 2).

**Fig. 3:**
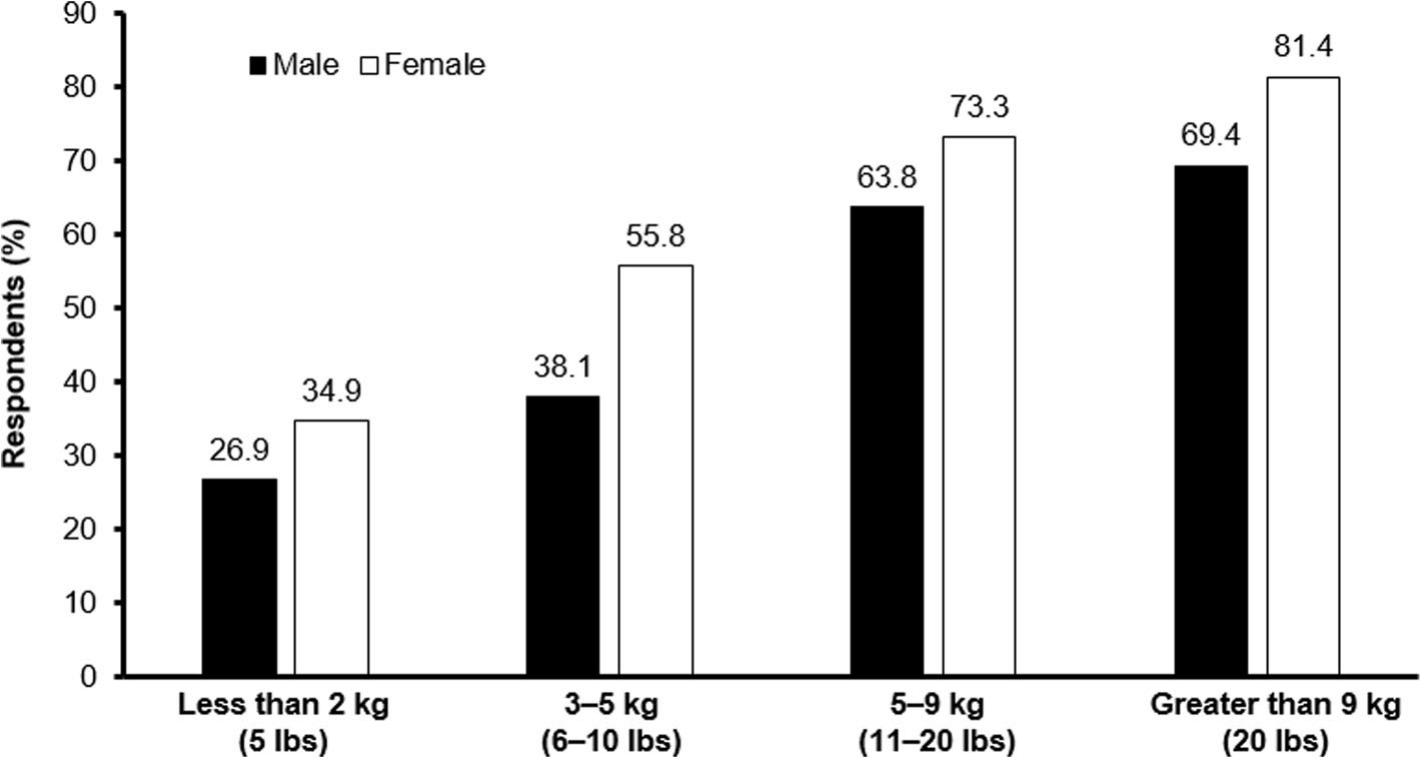
Patients who would not take a medication or who said their decision would be greatly influenced by the anticipated range of weight gain (by gender)*^,†^. *Data not included for patients who did not self-report gender (*n* = 4). ^†^210 patients (84%) surveyed had responses that did not change or increased across increasing categories of weight gain. 40 (16%) surveyed had inconsistent responses across weight gain categories. Source: Survey Question 3. One side effect of many medicines for schizophrenia is weight gain. How much would gaining weight influence your decision to take a medicine?

Across all ranges of weight gain, female patients rated the influence of weight gain higher in their decision to take medication than did male patients (Fig. 3). For an anticipated weight gain of 5.0–9.1 kg (11–20 lb), 73.3% of female patients, compared with 63.8% of male patients, indicated that this would influence their decision to take a medicine. A total of 81.4% of female patients, compared with 71.6% of male patients, reported that an anticipated weight gain of > 9 kg (> 20 lb) would influence their decision to take a medicine. Although patients with a BMI ≥ 27 kg/m^2^ viewed gaining weight as a more important side effect than those with a BMI < 27 kg/m^2^ (64.9% and 53.4% rated it “most important” or “very important,” respectively), the level of influence of different degrees of weight gain on the patient’s decision to take a medicine was similar.

When asked about their interest in new medications for the treatment of schizophrenia, 49.4% of patients expressed a likelihood they would try a new medicine (responded “very likely” or “likely”). The most common reasons for responding with an interest in a new medication were to see whether a new medication would have better efficacy and allow them to achieve their personal goals. Those who reported that they were “somewhat likely,” “unlikely,” “very unlikely,” or “not sure” about trying a new medication cited good efficacy of their current treatment or fear of unknown side effects as the rationale for their choice. Patients who considered gaining weight to be an important side effect were more likely to indicate an interest in trying a new medication (56% of patients who indicated that gaining weight was “most important” or “very important” were “very likely” or “likely” to try a new medication vs. 38% who reported gaining weight as “somewhat important” or “not important”).

## Discussion

From the present survey of patients with schizophrenia spectrum disorders, attitudes toward antipsychotic medication are influenced by both efficacy and side effect profile. The efficacy of medication therapy (ability to think clearly and reduce positive symptoms, such as voices and paranoia) was the most important factor for most patients. Side effects, including weight gain, were identified as important factors in determining willingness to take antipsychotic medications, with the level of influence on the decision to take a medicine increasing as anticipated weight gain increased – a finding that was particularly pronounced in female patients. Previous studies have highlighted the importance of weight gain as a cause for both patient distress and nonadherence to prescribed medications [10, 12, 13], as well as poor health outcomes [14]. Individual patient thresholds for side effects such as weight gain are therefore an important consideration when selecting medication or switching medication for schizophrenia.

There was variability in the perceived importance of side effects according to the baseline characteristics of the patients who responded, with female patients rating all side effects included in the survey as of greater importance than did male patients. In a previous patient survey designed to examine the relationship between self-reported side effects and adherence in schizophrenia, patient-reported side effects were strongly associated with self-reported nonadherence; only 42.5% of 876 patients with schizophrenia reported complete adherence to their antipsychotic medication [7]. Findings also suggest that, beyond efficacy and safety assessments, the success of treatment in schizophrenia depends on patient-reported tolerability to medications, a key factor impacting treatment adherence [15]. It is therefore important that clinicians working with patients who take antipsychotics adopt a mutually agreed decision-making framework where the selection of antipsychotic medication is guided by both efficacy and associated side effects, thereby addressing patients’ concerns, and permitting the development of an effective and well-tolerated treatment strategy [2, 16].

Given that the classification of antipsychotics into typical and atypical may not be a helpful distinction [17, 18], antipsychotic selection should be based on an individual risk-benefit assessment that evaluates the psychopathology, the patient’s medical comorbidities, and potential medication side effects. Inclusion of patient-reported outcomes in the design of clinical trials is required to ensure the development of treatment options that allow patients to achieve their personal goals [15, 19]. Determining and incorporating these patient goals and thresholds are of particular importance for clinical trial design in psychiatry, an area in which endpoints are based on subjective interpretation by trial investigators, patients, or both. Patient-reported outcomes can also be used to help inform the development of systems to reduce nonadherence to medication, such as medication reminder systems, to improve accuracy of administration, thereby improving patient outcomes [20, 21].

This study has several limitations. Because all data were garnered through self-reported responses to a cross-sectional survey, no baseline demographic characteristics and medical history were collected; hence, patients’ ages at diagnosis, current treatment, and treatment histories were not reported. Because age at diagnosis was not reported, it was not possible to determine the chronicity of schizophrenia among the survey respondents and the impact of duration of illness on attitudes towards antipsychotic medication. We recognize that this makes interpreting the study more difficult for clinicians treating patients with varying lengths of illness. However, we think the central theme that patient input should be considered when selecting antipsychotic medication treatment remains relevant. Not all subjective side effects were assessed in this short form, although the ones selected were guided by stakeholder input. The survey required patients to imagine the impact of a side effect they may or may not have experienced, which could have influenced the importance they attributed to the side effects. For example, patients who experienced extrapyramidal symptoms might have placed greater importance on those side effects because of first-hand experience in daily life. Patients’ perceptions of the importance of efficacy and side effects could also have been biased if they previously experienced inadequate treatment response or treatment switching. Conversely, by basing the study on a population of outpatients capable and willing to participate in a survey, bias could have been introduced toward patients experiencing reasonably good efficacy with their current treatments.

## Conclusions

When considering whether to take a medication, schizophrenia patients are influenced by many factors, including medication efficacy and side effects. It is important for clinicians to assess all concerns patients may have and to incorporate these into a comprehensive treatment plan designed to maximize adherence to the prescribed therapy.

## Additional file

Additional file 1: Schizophrenia, Patient Survey. (DOCX 20 kb)

## Abbreviations

BMI: Body mass index
CATIE: Clinical Antipsychotic Trials of Intervention Effectiveness
PANSS: Positive and Negative Syndrome Scale
