# Supplementary material for: Patient preferences concerning the efficacy and side-effect profile of schizophrenia medications: a survey of patients living with schizophrenia
Source: BMC Psychiatry. 2018 Sep 12;18:292. doi: 10.1186/s12888-018-1856-y (PMC6142379; doi:10.1186/s12888-018-1856-y)
Supplement: Supplementary file 1 — Schizophrenia, Patient Survey. (DOCX 20 kb) [file 12888_2018_1856_MOESM1_ESM.docx]

## Additional file – Schizophrenia, Patient Survey

Dear Potential Research Subject,

By completing the attached survey you are providing consent to participate in a research study. The purpose of this study is to collect information about patients’ opinions regarding medications used to treat schizophrenia. Your participation is entirely voluntary. If you agree to participate, you may choose not to answer any given questions, and you may withdraw your consent and discontinue your participation at any time. Thank you for your consideration of participating in this study.

**The following questions are about you**

1) You are a:

Male □ Female □

2) Your age is: ___ years old

3) Your height is: ___ feet ___ inches

4) Your current weight is: ___ pounds

**The following questions are about medicines that treat schizophrenia, schizoaffective disorder**

1) If there were a new medicine available to you that could treat symptoms of your schizophrenia, what qualities of this medicine would you find most helpful?

| Attributes of a new medicine | Most important | Very important | Somewhat important | Not important | I don’t have this problem |
| --- | --- | --- | --- | --- | --- |
| Makes the hallucinations or  paranoia go away | □ | □ | □ | □ | □ |
| Brings back my emotions or  ability to feel, smile or control my emotions | □ | □ | □ | □ | □ |
| Helps me think more clearly | □ | □ | □ | □ | □ |
| Has fewer side effects than the  one I take currently | □ | □ | □ | □ | □ |

2) Many current treatments for schizophrenia have side effects. How important are these side effects to you?

| Side effects of medications for schizophrenia | Most important | Very important | Somewhat important | Not important | I’ve never had this side effect |
| --- | --- | --- | --- | --- | --- |
| Feeling tired or drowsy | □ | □ | □ | □ | □ |
| Gaining weight | □ | □ | □ | □ | □ |
| Feeling restless or having  uncontrollable movements | □ | □ | □ | □ | □ |

3) One side effect of many medicines for schizophrenia is weight gain. How much would gaining weight influence your decision to take a medicine?

| Amount of weight you could gain | This wouldn't influence my decision at all | This would influence my decision a little | This would influence my decision a lot | I would not take this medicine |
| --- | --- | --- | --- | --- |
| Less than 5 pounds | □ | □ | □ | □ |
| 6–10 pounds | □ | □ | □ | □ |
| 11–20 pounds | □ | □ | □ | □ |
| Greater than 20 pounds | □ | □ | □ | □ |

4) When you are deciding whether to take a medicine that has been prescribed to you, how important is each of the following factors?

| Factors that influence your decision to take your medicines | Most important | Very important | Somewhat important | Not important |
| --- | --- | --- | --- | --- |
| How well the medication treats my schizophrenia | □ | □ | □ | □ |
| Side effects of medication | □ | □ | □ | □ |
| How easy the medication is to take | □ | □ | □ | □ |
| Somebody reminds me or gives me the medication | □ | □ | □ | □ |
| If I'm actively having symptoms | □ | □ | □ | □ |

5) How likely are you to try a new medicine for the treatment of schizophrenia?

| Very likely | Likely | Somewhat likely | Unlikely | Very unlikely | Not sure |
| --- | --- | --- | --- | --- | --- |
| □ | □ | □ | □ | □ | □ |

Please explain why. [Open text]

6) How likely are you to participate in a clinical research study (trial) investigating new medicine for the treatment of schizophrenia?*

| Very likely | Likely | Somewhat likely | Unlikely | Very unlikely | Not sure |
| --- | --- | --- | --- | --- | --- |
| □ | □ | □ | □ | □ | □ |

Please explain why. [Open text]

*Responses to survey question 6 were not included as they are not relevant to this report of patients’ medication preferences.
